# Aryl hydrocarbon receptor deficiency enhances astrocyte sensitivity to LPS-induced inflammation

**DOI:** 10.3389/fncel.2025.1653109

**Published:** 2025-11-14

**Authors:** Emmanuel Ojo, Shelley A. Tischkau

**Affiliations:** 1 Department of Pharmacology, Southern Illinois University School of Medicine, Springfield, IL, United States; 2 Department of Medical Microbiology, Immunology and Cell Biology, Southern Illinois University School of Medicine, Springfield, IL, United States

**Keywords:** lipopolysaccharide (LPS), astrocyte, cytokines, inflammation, aryl hydrocarbon receptor

## Abstract

The aryl hydrocarbon receptor (AhR) is a ligand-activated transcription factor linked to the control of immunological responses. Although AhR has been investigated in relation to lipopolysaccharide (LPS) peripheral inflammation, its role in LPS-induced, astrocyte-mediated inflammation *in vivo* is unknown. This study explores the effect of AhR deletion on astrocyte reactivity and neuroinflammation responses to lipopolysaccharide (LPS). The results show that AhR loss aggravates LPS-induced inflammatory responses using a AhR germline knockout (AhRKO) mouse by increasing pro-inflammatory cytokines levels (TNF-α, IL-1β) and inducible nitric oxide synthase (iNOS) in both primary astrocyte cultures and the mouse hippocampus. Morphologically, astrocytes and microglia from AhRKO mice show increased soma size following LPS injection, suggesting increased glial activation. In addition, AhRKO mice displayed more severe weight loss and locomotor impairment behaviorally following a single systemic LPS injection. Elevated nuclear translocation of NF-κB p65 in AhR-deficient astrocytes provides a potential mechanism for elevated pro-inflammatory signaling. These results emphasize an immunomodulatory role for AhR in reducing astrocyte-driven inflammation and identify AhR as possible therapeutic target for neurodegenerative illnesses linked with neuroinflammatory responses.

## 1 Introduction

Astrocytes are morphologically complex cells that are diverse and heterogenous across several brain regions (Oberheim et al., 2012; Vidal-Itriago et al., 2022; Zhou et al., 2019). These cells are reported to be involved in neuroinflammation-linked disorders, such as Alzheimer’s disease and Parkinson’s disease (Kwon and Koh, 2020; Zhao et al., 2024). Mouse neuroinflammatory models have previously been developed to study the response of glial cells to toxins or genetic alterations (da Silva et al., 2024; Nazem et al., 2015; Schwab et al., 2010). Lipopolysaccharide (LPS), injected either systemically or intracerebrally (Qin et al., 2007; Zhao et al., 2019), serves as a common experimental model for inducing CNS inflammation and mirrors the neuroinflammatory signature observed in many neurodegenerative diseases. LPS injection activates astrocytes and microglia in mice (Brandi et al., 2022; da Silva et al., 2024; Norden et al., 2016). Although cytokines, chemokines and other genes linked to astrocyte or microglia inflammatory responses have been used as biomarkers to characterize glial reactivity in the LPS model, morphological changes of glia cells are also recognized as structural features of neuroinflammation (Diaz-Castro et al., 2021; Guerrero-Carrasco et al., 2024). Activated astrocytes exhibit anatomical hypertrophy, which includes increased soma size and thickening of main branches, as well as increased overall branching (Gomes et al., 2024; Pekny and Pekna, 2014). Also, microglial cells switch from a homeostatic ramified shape to a unramified ameboid shape following either systemic or intracerebral LPS administration (Kim et al., 2024).

Aryl hydrocarbon receptor, a ligand activated transcription factor, is expressed in both astrocytes and microglia, and has been implicated in age-related disease processes (Rothhammer et al., 2021; Wheeler et al., 2019; Zhou et al., 2021). Heighted inflammatory responses in peripheral tissues such as lungs and liver have been reported in AhR-deficient mice after systemic LPS injection (Sekine et al., 2009; Wu et al., 2011). Moreover, AhR signaling also serves as a crucial regulator of immunological response to LPS by microglia in various *in vitro* systems (Khan et al., 2021; Lee et al., 2015). AhR-mediated astrocyte functionality in response to CNS inflammatory stimulants such as LPS both *in vitro* and *in vivo*, under conditions that more closely represents physiological responses of glia cells in the brain, requires further investigation. Therefore, the aim of this study is to determine how AhR mediates astrocyte cell reactivity in the context of their native environment during inflammatory conditions using changes in morphological features, cytokines, and changes in behavior, as indicators of the inflammatory response.

## 2 Materials and methods

### 2.1 Animals

Protocols for animal utilization received approval from the Institutional Animal Care and Use Committee at Southern Illinois University School of Medicine and were executed in compliance with the Guide for the Care and Use of Laboratory Animals as established by the National Institutes of Health. Experiments utilized 9–10-week-old male C57BL/6J, germ line AhR null (AhRKO) Bradfield strain (Schmidt et al., 1996), obtained from the Jackson Laboratory (Bar Harbor, ME) and bred at the Southern Illinois University School of Medicine animal facilities. All animals were housed in groups and entrained to a control 12:12 h light: dark schedule with food and water provided ad libitum.

### 2.2 Primary cell cultures

Primary hippocampus cell cultures were established from postnatal day 0–1 C57BL6/J and AhRKO pups using methods previously described by Gutiérrez-Vázquez and Quintana, 2022. Hippocampi were dissected and dissociated using papain (EC 3.4.22.2; Brain Bits) treatment, followed by trituration with sterile glass pipettes. Hippocampal cells were cultured using NbAstro medium (Brain Bits) (Neurobasal, 10% horse serum, Glutamax, and 1% penicillin/streptomycin) at 37 °C with 5% CO_2_ for days *in vitro* (DIV) 10–14. The cell culture media was changed the day following seeding and subsequently every 3 days. Upon reaching approximately 90% confluence, adherent astrocytes were detached using Trypsin (0.25%) (Cat: 15050057; Gibco, United States) and subsequently replated, maintaining them in serum-free media for an additional 1–2 days before exposure to 250 ng/ml of LPS from *Escherichia coli* 0127: B8 (Sigma-Aldrich Cat: L3129). Approximately 96% of cultured astrocytes were stained with polyclonal glial fibrillary acidic protein (GFAP) as previous shown in our lab (Ojo et al., 2025).

### 2.3 LPS injection

Male mice aged 9–10 weeks received a single intraperitoneal injection of 5 mg/kg LPS (from Escherichia coli 0127: B8 Sigma-Aldrich Cat: L3129) or PBS. LPS was solubilized in PBS to achieve a final concentration of 1 mg/ml, and the injection volume was calculated according to body weight. Twenty-four hours post-injection, some mice were euthanized, and the brain was harvested for histological and molecular analysis while the other mice were utilized for behavioral studies.

### 2.4 RNA extraction and qPCR

2–2.5 × 10^6^ astrocytes per T-25 flask, and mouse hippocampi were lysed in Trizol (Fisher Scientific, Hampton, NH, United States), and RNA was isolated using the extraction protocol. cDNA was synthesized and SYBR green-based real-time reverse transcriptase PCR was performed on a Quant-Studio real-time PCR system. Gene expression values were normalized using GAPDH as the housekeeping gene, and relative mRNA levels were determined using the ΔΔCt method. The primer sequences for real-time PCR are provided in Table 1.

**TABLE 1:** List of primers.

Genes	Forward primer sequence	Reverse primer sequence
TNF-α	5′-CCA CCA CGCTCT TCT GTCTAC-3″	5′-AGG GTC TGG GCCATA GAA CT-3′
IL-β	5′-AGATGAAGGGCTGCTTCCAAA-3′	5′-GGAAGGTCCACGGGAAAGAC-3′
IL-10	5′-AGGCGCTGTCATCGATTTCT-3′	5′-ATGGCCTTGTAGACACCTTGG-3′
iNOS	5′-ACATCGACCCGTCCACAGTAT-3′	5′-CAGAGGGGTAGGCTTGTCTC-3′
CCL2	5′-CCACTCACCTGCTGCTACTCAT-3′	5′-TGGTGATCCTCTTGTAGCTCTCC-3′
C3	5′-AGCTTCAGGGTCCCAGCTAC-3′	5′-GCTGGAATCTTGATGGAGACGC-3′
S100A10	5′-CCAGGTTTCGACAGACTCTTC-3′	5′-CCGTTCCATGAGCACTCTC-3′
GAPDH	5′-ATGGTGAAGGTCGGTGTGAAC-3′	5′-TGTAGTTGAGGTCAATGAAGG-3′

### 2.5 Immunofluorescence (IF)

A total of 50,000 hippocampal astrocytes per 4-well glass slide (1.7 cm^2^ per well) were washed with PBS and subsequently fixed with 4% paraformaldehyde (PFA) for 20 min at room temperature. Cells were permeabilized with PBST (0.1M PBS with 0.25% TritonX-100), followed by a 1-h incubation in a solution of 10% normal goat serum and 1% bovine serum albumin (BSA). After incubation, primary antibodies were applied at 4 °C overnight. Cells were rinsed with PBST (0.1M PBS with 0.25% TritonX-100) before incubation with secondary antibodies in the dark at room temperature for 2 h. The cells were subsequently washed in PBST and were cover slipped with ProLong™ Gold antifade reagent containing DAPI. Staining was examined using confocal microscopy, and number of cells with p65 nuclear translocation were counted with National Institute of Health Image J Software 1.48 (RRID:SCR_003070) by accessing the Dapi/p65 overlay in astrocyte cells. Primary antibodies included: Chicken polyclonal glial fibrillary acidic protein antibody (1:1000 biosensis Catlog:C-1373-50), Rabbit monoclonal anti-NF-KB p65 antibody (1:400 Cell signaling Catlog:#8242) while secondary antibodies used were Goat anti-chicken IgY H&L (Alexa Fluor^®^ 594) 1:1000, Goat anti-rabbit IgG (H+L) (Alexa Fluor^®^ 488) 1:1000.

For tissue staining, 20 μm hippocampal sections were cut on a cryostat (Model HM525 NX, ThermoFisher Scientific). Serial sections were extracted from every sixth section of the hippocampus. Hippocampal slices were subjected to immunofluorescence using a chicken polyclonal glial fibrillary acidic protein (GFAP) antibody at a dilution of 1:500 and a rabbit polyclonal anti-IBA1 antibody at a dilution of 1:500. Sections were permeabilized in PBST (0.1M PBS with 0.25% Triton X-100) and subsequently washed three times for 10 min each in sodium borohydride in PBS (1 mg/ml) for antigen retrieval. Slices were then subjected to another wash with PBST and subsequently incubated in a solution of 10% normal goat serum and 1% BSA for 1 h. Primary antibodies were then applied to the hippocampal sections in a humid chamber at 4 °C. On the subsequent day, slices were washed in PBST and incubated with the secondary antibody (goat anti-rabbit IgG H&L and goat anti-chicken IgY at 1:1000 dilution) for 2 h. The sections were subsequently washed in PBST and were covered with DAPI-containing ProLong™ Gold antifade reagent. Z-stack images of five optical slices at 1 μm were obtained using a 40x oil immersion objective (numerical aperture 1.3) with adjusted pinhole size of 0.81 AU on a Zeiss LSM800 confocal microscopy system at 1,024 × 1,024 pixels. For astrocyte and microglia soma size, 40X z stacks confocal images were imported into the imaris software and a 3D surface were built around the immunofluorescence z-stacks based on GFAP and IBA-1 staining of the cells, total soma size was determined and exported.

### 2.6 Behavioral battery

Mice were evaluated using a behavioral battery to explore locomotion and memory function. All behavioral assessments were conducted under low red-light conditions (15–20 lux) during the dark phase, 24 h following injection of LPS. Video tracking and automated analysis (Noldus EthovisionXT v17.5) were employed to assess animal behavior in the open field, Y-maze, and Novel Object tests.

#### 2.6.1 Y-maze

Using a Y-shaped maze with three arms each 35 cm long and 5 cm wide, each diverging from a central point at 120° angles, with walls 20 cm high, short-term working memory was measured. For 10 min, mice were allowed to explore the novel Y-maze while their entry was recorded as the moment the mouse’s body passed into an arm. The total number of arm entries and spontaneous alternations (entry into three distinct arms in successive choices) were recorded and expressed as a percentage of alternation (number of alternations divided by total number of arm entries).

#### 2.6.2 Open field test

Mice were assessed in an open field (40 × 40 cm box) for 30 min to measure motor function. Mice were monitored in an open field to assess total voluntary distance traveled, duration spent in the center (20 × 20 cm) area equidistant from all edges, total entries into the center. The center point of the body was utilized to ascertain the mouse’s location within the arena.

#### 2.6.3 Novel Object Recognition

The Novel Object Recognition (NOR) assay was employed to evaluate long-term memory in mice by quantifying the exploration duration of a novel object relative to a familiar object within a testing arena. The mice were initially positioned in the NOR testing arena (40 × 40 cm box) for a 30-min habituation period. Twenty-four hours post-habituation, two similar objects were introduced into the testing arena, and mice were permitted to explore the objects for 10 min; this phase is referred to as the training day. Following a 24-h inter-session interval, mice were reintroduced into the novel object test arena for 10 min, where they encountered one familiar object from the prior training day alongside a novel object to assess memory retention. Novelty preference (% time) was determined by the ratio of total time spent with the novel object to the total time spent with both objects.

### 2.7 Statistical analysis

Prism (GraphPad Software, Inc., La Jolla, CA; RRID:SCR_ 002798) software was used for statistical analyses. Data are presented as mean ± SEM. Unless otherwise stated, all cell culture experiments were repeated at least three times, from separate dissections. One-way or two-way ANOVA with Tukey’s post-hoc tests were utilized to identify significant differences between groups, where appropriate. For all behavioral assays, both automated and manual data were acquired from EthoVision XT 17.5 and measurements were analyzed using two-way ANOVA with Tukey’s post-hoc tests. Statistical significance was defined as *p* < 0.05.

## 3 Results

### 3.1 AhR deletion increases pro-inflammatory cytokine levels in astrocyte cell culture and hippocampus of LPS treated mice

Prior research has shown that AhR activation exerts immunosuppressive effects on LPS-induced proinflammatory cytokine production in primary microglial cultures (Brandi et al., 2022; Guerrero-Carrasco et al., 2024; Kim et al., 2024). Therefore, we determined whether AhR activation elicits a comparable immunomodulatory response in astrocytes under conditions of acute LPS-induced neuroinflammation in an *in vitro* system.

**FIGURE 1:**
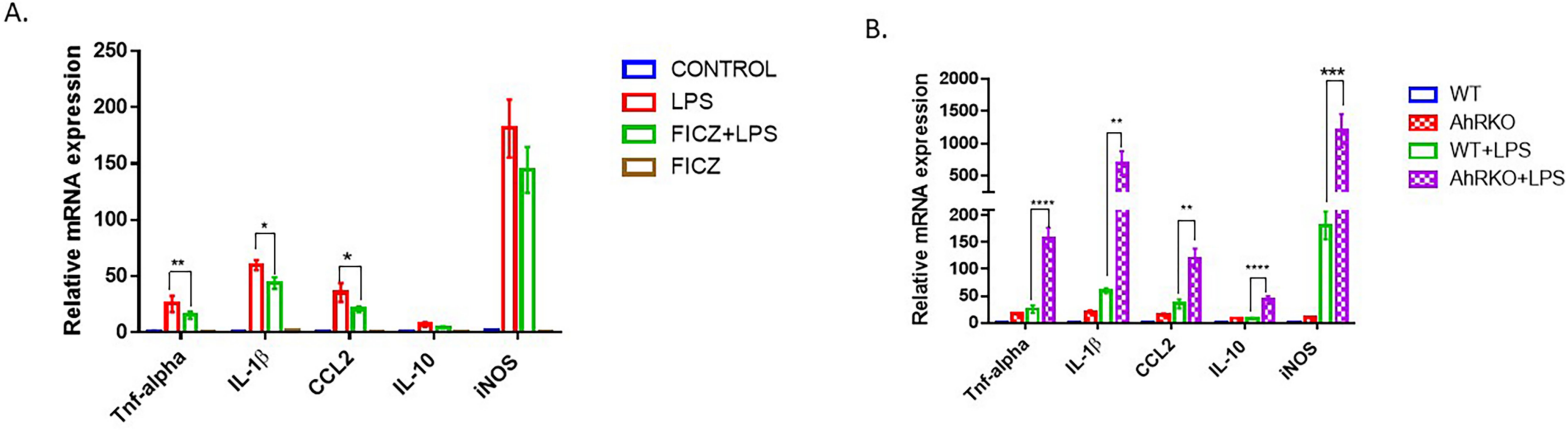
Aryl hydrocarbon receptor (AhR) deletion increases lipopolysaccharide (LPS)-induced inflammatory response in primary astrocyte cell culture. **(A)** Fold change of mRNA levels relative to control expression levels of inflammatory cytokines in hippocampal astrocytes pretreated with FICZ (250 nM) followed by LPS for 24 h. Data represent mean ± S.E.M, *n* = 3–4 independent biological replicates. *P < 0.05, **P < 0.01 by One-way ANOVA with Tukey’s *post hoc* comparison. **(B)** Fold change of mRNA levels relative to wild type expression levels of inflammatory cytokines in AhR-deficient astrocytes treated with by LPS for 24 h. Data represent mean ± S.E.M, *n* = 3–4 independent biological replicates. **P < 0.01,***P < 0.001, ****P < 0.0001 by Two-way ANOVA with Tukey’s *post hoc* comparison.

Cytokine profiling was performed to assess the impact of AhR signaling on LPS-induced astrocyte reactivity *in vitro*. LPS treatment elevated the transcript levels of various pro-inflammatory cytokines (TNF-α, IL-1β, and CCL2). Furthermore, pretreatment of astrocytes with the AhR agonist, FICZ, suppressed the LPS-induced increase in TNF-α [F _(3,12)_ = 9.392, *p* = 0.0018], IL-1β [F _(3,10)_ = 83.24, *p* < 0.0001], CCL2 [F _(3,12)_ = 14.92, *p* = 0.0002, one-way ANOVA] pro-inflammatory cytokines (Figure 1A). Previous studies from our lab have confirmed that AhR is activated by FICZ by assessing downstream targets Cyp1a1 in our astrocyte culture (Ojo et al., 2025). In contrast, AhRKO-derived astrocyte cell cultures treated with LPS exhibited significant increases in the transcript levels of TNF-α [F _(1,12)_ = 32.79, *p* < 0.0001], IL-1β [F _(1,12)_ = 10.86, *p* = 0.0064], CCL2 [F _(1,12)_ = 12.09, *p* = 0.0046, two-way ANOVA] when compared to LPS-treated wild type controls. To assess anti-inflammatory cytokines, IL-10 transcript levels were evaluated. Surprisingly AhRKO-derived astrocytes also demonstrated more robust elevation of IL-10 mRNA levels 24 h following LPS treatment [F _(1,11)_ = 11.43, *p* = 0.0061, two-way ANOVA] (Figure 1B). In addition to elevated pro-inflammatory cytokines, LPS also enhanced the levels of inducible nitric oxide synthase (iNOS) in our astrocyte cell culture system. Although these levels were not diminished by FICZ pretreatment, iNOS transcript levels were elevated in AhR-deficient astrocyte cells [F _(1,11)_ = 12.45, *p* = 0.0047, two-way ANOVA]. We observed that untreated AhR-deficient astrocyte cultures exhibited a trend toward an increase in proinflammatory cytokines compared to untreated wildtype astrocyte cultures, this indicates that the absence of AhR in astrocyte cells, in the absence of inflammatory stimuli, renders these cells susceptible to inflammatory processes (Figure 1B).

**FIGURE 2:**
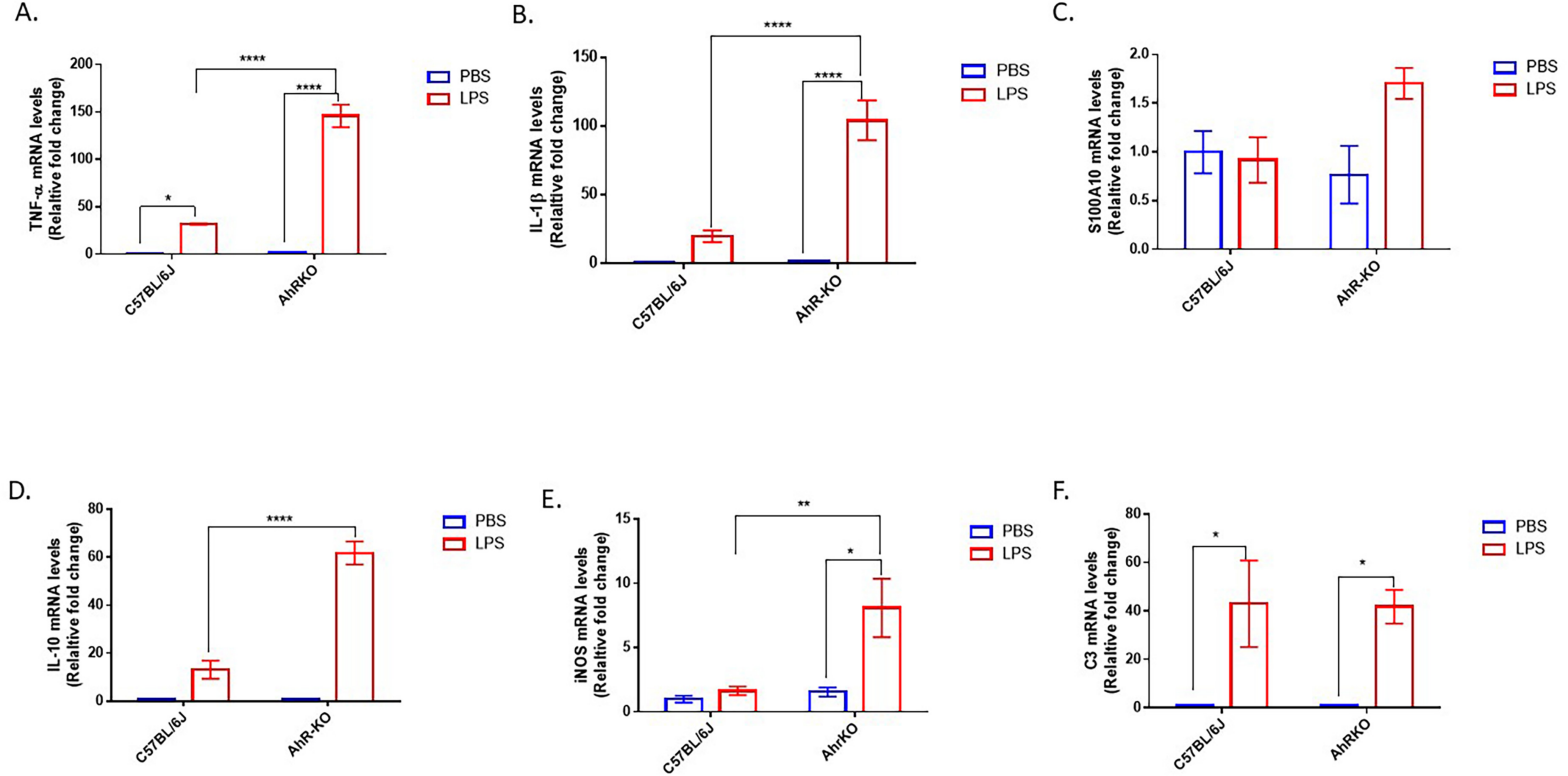
Aryl hydrocarbon receptor germline knockout (AhRKO) mice exhibit enhanced pro-inflammatory cytokine levels in the hippocampus. **(A–F)** Fold change of mRNA levels relative to wild type expression levels of inflammatory cytokines in the hippocampus of PBS and lipopolysaccharide (LPS) treated mice 24 h after injection. Data represent mean ± S.E.M, *n* = 3–4 animals per group. *P < 0.05, **P < 0.01, ****P < 0.0001 by Two-way ANOVA with Tukey’s *post hoc* comparison.

*In vivo*, LPS treatment produced significantly higher transcript levels for various pro-inflammatory cytokines (TNF-α and IL-1β). However, similar to the *in vitro* astrocyte culture, the hippocampus of LPS-treated AhRKO mice exhibited significantly increased levels of TNF-α [F _(1,8)_ = 88.82, *p* < 0.0001, two-way ANOVA] and IL-1β [F _(1,12)_ = 28.31, *p* = 0.0002, two-way ANOVA] compared to LPS-treated wild-type controls (Figures 2A, B). Notably, increased levels of anti-inflammatory cytokine-associated genes (S100A10 and IL-10) [F _(1,11)_ = 42.30, *p* < 0.0001, two-way ANOVA] were also observed in LPS-treated AhRKO mice (Figures 2C, D). We also assessed the levels of iNOS (marker for oxidative stress) and C3 (marker for reactive glia cells) in the hippocampus of LPS injected mice. iNOS mRNA was substantially increased in LPS-injected AhRKO mice relative to LPS injected wild-type mice [F _(1,12)_ = 6.101, *p* = 0.0295, two-way ANOVA], with no significant changes in C3 mRNA [F _(1,11)_ = 0.5071, *p* = 0.4912, two-way ANOVA] (Figures 2E, F). Altogether, these results suggest that deletion of AhR heightened the immune response to inflammatory stimuli, as observed both *in vitro* in primary astrocyte cultures and *in vivo* in extracts derived from whole hippocampus.

### 3.2 AhRKO exacerbates the morphological glial cell inflammatory response to LPS *in vivo*

**FIGURE 3:**
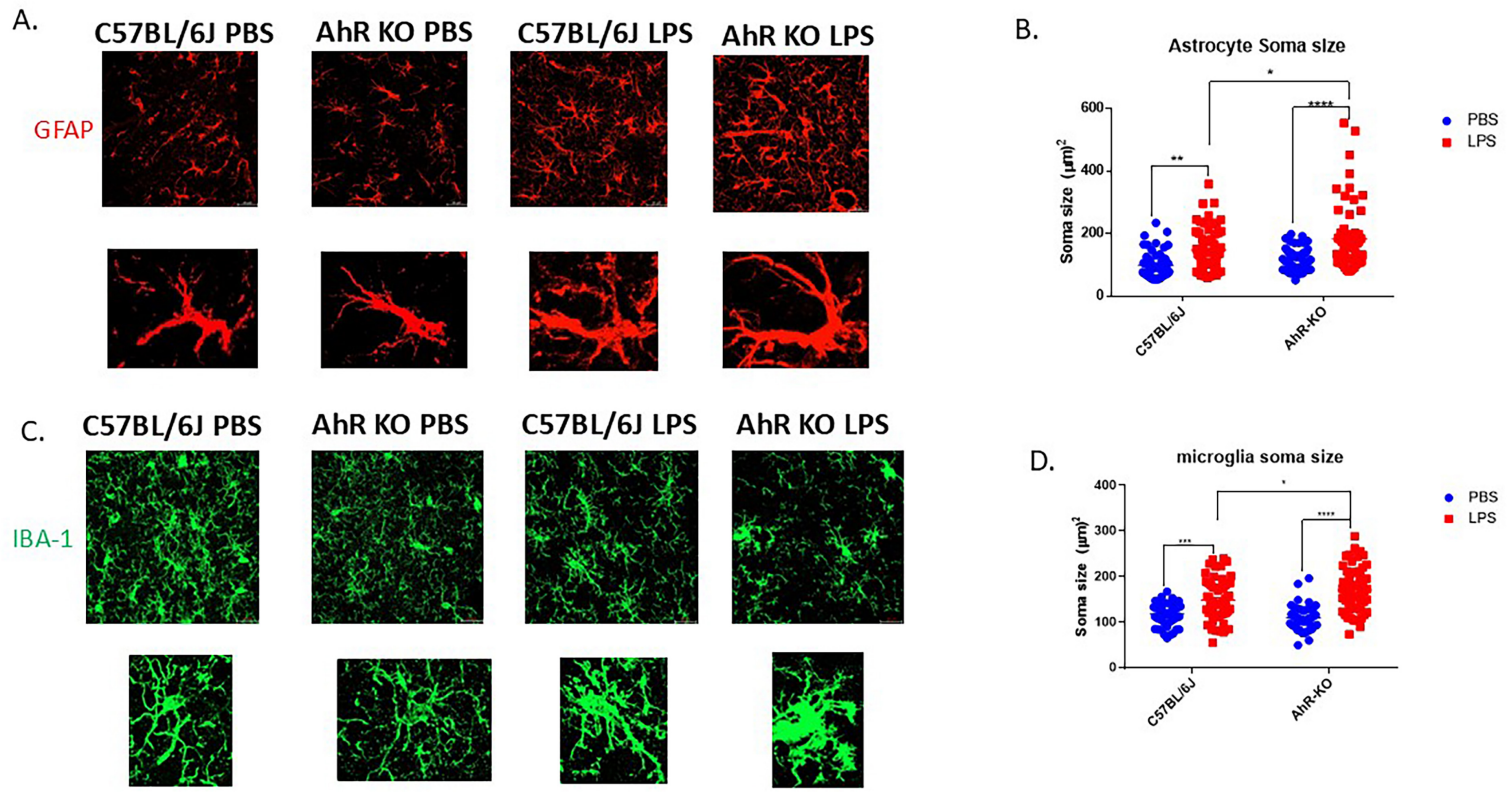
Aryl hydrocarbon receptor (AhR) deletion exacerbates glial cell inflammatory response to lipopolysaccharide (LPS) *in vivo*. **(A)** Representative immunofluorescence stain of astrocytes in the CA1 region of the hippocampus 24 h post-LPS injection **(B)** Quantification of astrocyte soma size (μm^2^), total of 50–60 astrocytes per group. Data represent mean ± S.E.M, *n* = 4 animals per group. *P < 0.05, **P < 0.01, ****P < 0.0001 by Two-way ANOVA with Tukey’s *post hoc* comparison. All images taken at 40 × magnification, scale bar = 20 μm **(C)** Representative immunofluorescence stain of astrocytes in the CA1 region of the hippocampus 24 h post-LPS injection. **(D)** Quantification of microglial soma size (μm^2^), total of 50–60 microglia per group. *P < 0.05, **P < 0.01, ***P < 0.001, ****P < 0.0001 by Two-way ANOVA with Tukey’s *post hoc* comparison. All images taken at 40 × magnification, scale bar = 20 μm

To assess the effect of AhR deficiency on the inflammatory response of glial cells in their native environment, structural alterations in astrocytes and microglia were evaluated following a single, acute systemic injection of LPS. In this neuroinflammatory model, a substantial population of hippocampal microglia and astrocytes exhibit inflammatory-induced morphological alterations 24 h after a single LPS injection. LPS generated an increase in astrocyte soma size compared to the PBS-treated group [F _(1,226)_ = 36.61, *p* < 0.0001, two-way ANOVA]; furthermore, these structural alterations were exacerbated in AhRKO mice astrocytes, which had more hypertrophic characteristics than LPS-treated wild-type controls (Figures 3A, B). Comparatively, microglia in AhRKO animals also exhibited a higher inflammatory response, shown by a larger soma size than wild-type controls treated with LPS [F _(1,208)_ = 7.556, *p* = 0.0065, two-way ANOVA] (Figures 3C, D). These results further support the idea that AhRKO mice exhibit heightened sensitivity to LPS treatment in the brain.

**FIGURE 4:**
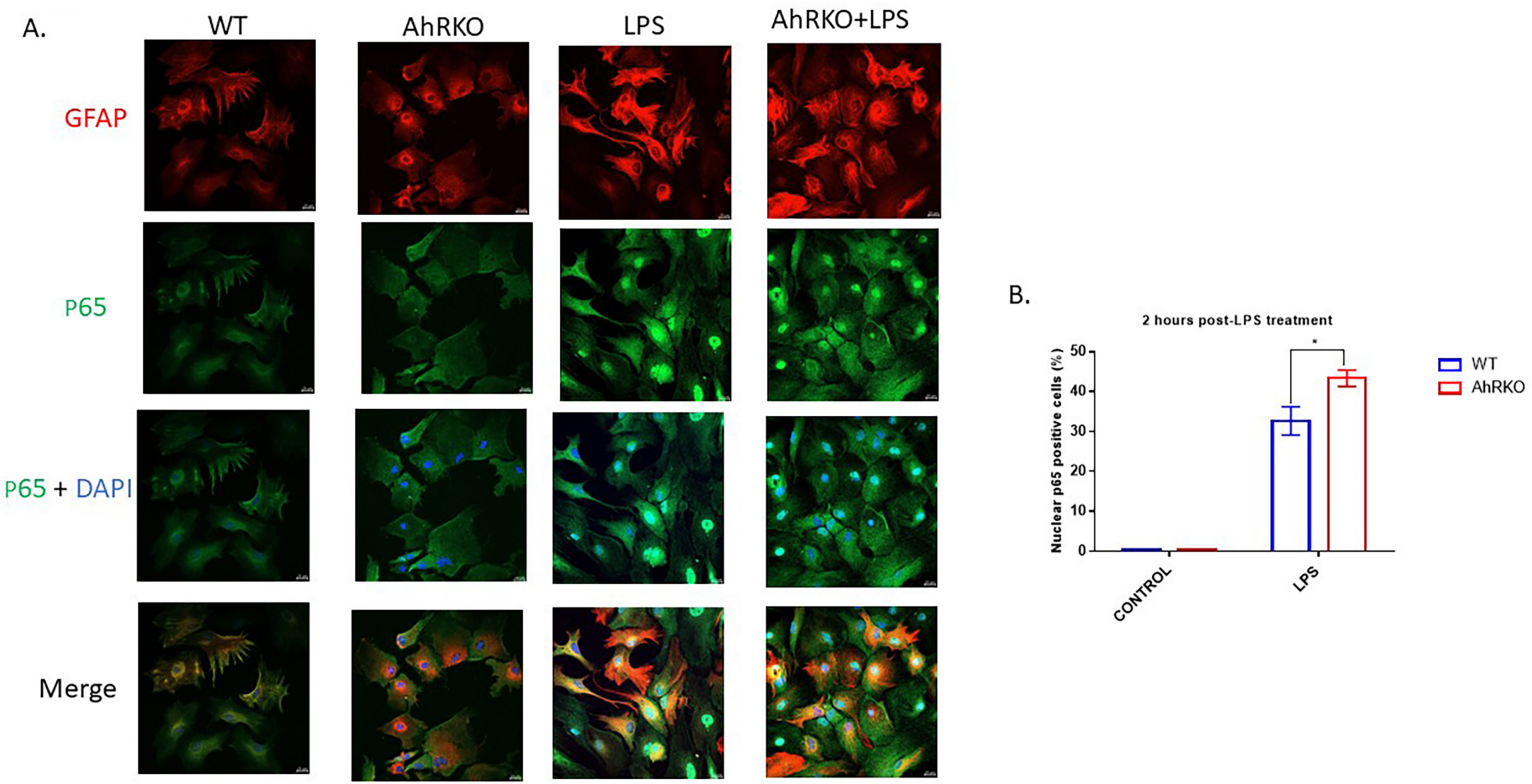
Aryl hydrocarbon receptor (AhR) deletion induces astrocyte cytokine expression through p65 NF-κB activation. **(A)** Representative images of primary astrocyte cells co-stained with glial fibrillary acidic protein (GFAP) (Red), p65 NF-κB (green) and dapi (blue). **(B)** Quantification of p65 positive cells in the nucleus of astrocytes following lipopolysaccharide (LPS) treatment. Data represents mean ± S.E.M, *n* = 4 independent biological replicates.*P < 0.05, by Two-way ANOVA with Tukey’s *post hoc* comparison. Images taken at 40 × magnification with scale bar = 20 μm from five different fields for each well.

To examine the signaling pathway by which AhR interacts to reduce the release of pro-inflammatory cytokines in astrocytes cultures after inflammatory stimulation, p65 NF-κB nuclear translocation was assessed 2 h following LPS treatment. LPS promoted more nuclear accumulation of p65 NF-κB in AhR-deficient astrocytes relative to the wild type astrocytes [F _(1,12)_ = 6.804, *p* = 0.0229, two-way ANOVA] (Figures 4A, B). Thus, from these results AhR activation might be interfering with NF-κB signaling to exert an immunosuppressive effect on astrocytes in response to LPS.

### 3.3 Behavioral alterations induced by systemic LPS administration are amplified in AhRKO mice

**FIGURE 5:**
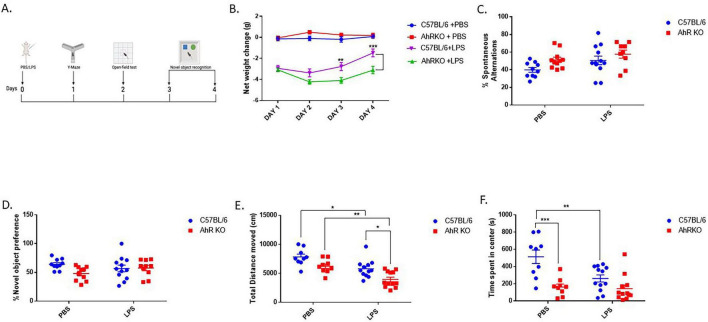
Aryl hydrocarbon receptor germline knockout (AhRKO) mice exhibited amplified behavioral changes following systemic lipopolysaccharide (LPS) treatment. **(A)** Schematic showing the overview of behavioral test design **(B)** Quantification of changes in body weight over 4 days following a single I.P injection of LPS **(C)** Percentage number of spontaneous alternations during Y-maze test 24 h post LPS injection. Data represent mean ± S.E.M, *n* = 9–12 animals per group **(D)** Duration spent with novel object during testing phase Data represent mean ± S.E.M, *n* = 10–12 animals per group **(E)** Total distance moved within 30 min period of open field exploration **(F)** Total time spent (s) at the center of the open field box during exploration. Data represent mean ± S.E.M, *n* = 9–12 animals per group. *P < 0.05, **P < 0.01, ***P < 0.001 by Two -way ANOVA with Tukey’s *post hoc*.

The impact of AhR deficiency on LPS-induced behavioral alterations were determined by assessing weight changes, cognitive impairment, and locomotor activity subsequent to systemic LPS injection (Figure 5A). LPS treatment resulted in a significant decrease in body weight compared to PBS injection throughout four days of weight assessment in mice; however, LPS-injected AhRKO animals exhibited more pronounced weight loss and resistance to returning to the normal weight range by Day 4 post-LPS injection [F _(9,182)_ = 3.268, *p* = 0.0010, two-way ANOVA] (Figure 5B). No significant cognitive differences were found in the Y-maze [F _(1,38)_ = 0.3016, *p* = 0.5861, two-way ANOVA] or Novel Object test [F _(1,38)_ = 3.358, *p* = 0.0747, two-way ANOVA] between the PBS and LPS-treated mice (Figures 5C, D). In the open field test, LPS administration reduced the overall distance traveled by the mice in comparison to those treated with PBS [F _(1,38)_ = 15.51, *p* = 0.0003, two-way ANOVA]. However, the diminished locomotor activity was more noticeable in the AhRKO mice administered LPS [F _(1,38)_ = 0.07172, *p* = 0.7903, two-way ANOVA] (Figure 5E). PBS-treated AhRKO mice also spent less time in the center of the open field box compared to PBS-treated wild-type mice, which is an indication of anxiety-like behavior displayed by AhRKO mice [F _(1,38)_ = 21.44, *p* < 0.0001, two-way ANOVA] (Figure 5F). Overall, these findings indicate that the deletion of AhR in mice amplifies acute behavioral alterations triggered by systemic LPS administration, particularly locomotor activity and body weight.

## 4 Discussion

A key mechanism by which the astrocytes participate in inflammatory responses during pathological conditions is by release of pro-inflammatory cytokines, chemokines, and other neurotoxic factors that promote neuronal dysfunction and synaptic loss (Kwon and Koh, 2020; Lee et al., 2023). This study demonstrated that AhR deletion intensifies inflammatory responses in astrocytes after LPS-induced systemic inflammation, which builds upon evidence from previous studies reporting hyper-responsiveness of AhR knockout mice to LPS, and immunosuppressive effects of AhR activation in immune cells (Kimura et al., 2009; Sekine et al., 2009; Zhu et al., 2018). The current study adds to the understanding of AhR signaling in regulating neuroinflammation through the immune responses of glial cells, specifically by modifying the morphology and cytokine expression of astrocytes and microglia in an *in vivo* system, potentially through regulation of NFkB.

Lipopolysaccharide injection is a common model for generating CNS inflammation; it mirrors neuroinflammatory signatures observed in neurodegenerative diseases (Cunningham et al., 2009; Deng et al., 2020; Lopez-Rodriguez et al., 2021). Multiple investigations have clearly demonstrated the activation of astrocytes and microglia subsequent to LPS injection in mice (Brandi et al., 2022; da Silva et al., 2024; Norden et al., 2016). While the molecular characteristics of glial reactivity are frequently used as outputs to define neuroinflammatory signatures, the dynamic and heterogenous morphological changes can also be used as markers of inflammation in various disease states (Kim et al., 2024; Li et al., 2023; Yakovlev et al., 2024). This study demonstrated that a single, systemic dose of LPS elicited profound astrocytic hypertrophic alterations in the mouse hippocampus, which is consistent with previously reported characteristics of activated astrocytes (Agnew-Svoboda et al., 2022; Diaz-Castro et al., 2021; Xingi et al., 2023). Moreover, the impact of LPS on molecular markers and morphological changes was markedly intensified AhR-depleted animals. Similarly, microglia in AhRKO animals also showed an exaggerated inflammatory response, as evidenced by increased soma size. The heightened morphological changes of glial cells to inflammatory stimuli seen in the brain of AhRKO mice indicates that the presence of AhR signaling during neuroinflammation may function as a critical checkpoint for modulating glial reactivity in the brain. Consistent with prior studies that identified AhR signaling as an immunological regulator in peripheral systems and immune cells, our studies corroborate this idea, and extends these findings to specific astrocytes and microglia within the brain. The presence of AhR signaling limits the activation of glial cells in response to inflammatory signals, thereby protecting the brain from intensified inflammatory responses following acute exposure to LPS. This serves as a foundational link that elucidates the function of AhR in regulating glial cells, particularly astrocyte responsiveness, within the framework of LPS-induced inflammation in the brain. Numerous immune cytokines have been associated with the onset and pathogenesis of various neuroinflammatory diseases. During the initiation and progress of neuroinflammation, reactive glial cells experience molecular alterations, including the secretion of proinflammatory cytokines such as IL-1β and TNF-α. The release of these cytokines in the brain promotes amplification of neuroinflammatory signaling and sustains the activation of glial cells, thereby contributing to chronic neuroinflammatory processes. AhR signaling contributes to shaping the transcriptional response of immune cells to inflammatory stimuli by binding to the DNA as a transcriptional factor to influence cytokines production (DiNatale et al., 2010; Ishihara et al., 2019; Kerkvliet, 2009). In our *in vitro* LPS model, AhR-deficient astrocytes had increased proinflammatory cytokine levels. The pro-inflammatory cytokine profile results observed in AhR-deficient astrocytes are consistent with previous studies utilizing primary microglial cultures, therefore indicating comparable AhR activation effects in both astrocytes and microglia immunological responses following LPS stimulation. Nevertheless, studies employing astrocyte–microglia co-culture systems are warranted, as such *in vitro* models would more accurately recapitulate the physiological neuroinflammatory cytokines responses observed *in vivo*, where astrocyte–microglia crosstalk plays a critical role in shaping inflammatory dynamics. Similarly, the hippocampus of AhRKO mice administered LPS systemically also demonstrated elevated production of inflammatory cytokines, such as TNF-α and IL-1β, thus providing more evidence that AhR regulates brain immune responses. However, we cannot exclude a potential contribution from AhR depletion in peripheral immune cells, as this study employed germ line AhR depleted mice. Peripheral immune cells may potentially affect the response of astrocytes or microglia to inflammatory stimuli, particularly when the blood-brain barrier has been compromised (Barton et al., 2019; Peng et al., 2021; VanHook, 2024). Thus, future experiments studying the response of astrocyte cells to inflammatory stimuli in mice with astrocyte-specific AhR deletion using efficient gene targeting strategies is still necessary.

A potential mechanism to explain how deletion of AhR increases proinflammatory cytokines in astrocytes after LPS is through interactions with NF-κB signaling, a major regulator of proinflammatory cytokine production in glia cells (Anilkumar and Wright-Jin, 2024; Dresselhaus and Meffert, 2019). In this study, enhanced NF-κB p65 translocation was observed in LPS-treated AhR-deficient astrocyte cultures, which is consistent with previous studies (Lin et al., 2022; Rothhammer et al., 2016). Surprisingly, we also observed an elevation of the anti-inflammatory cytokines IL-10 and S100A10 in the brains of AhRKO mice and in astrocyte cell cultures derived from AhRKO mice treated with LPS. When inflammatory stimuli activate astrocytes and microglia, elevated levels of IL-10 and S100A10 mostly suggest enhanced neuroprotective characteristics (King et al., 2020; Shanaki-Bavarsad et al., 2022). However, based on these findings, we speculate that these effects observed in LPS-treated AhRKO mice may result from compensatory mechanisms employed by neuroprotective glial phenotypes attempting to counterbalance the increased pro-inflammatory response (Barsig et al., 1995). While AhR activation by FICZ exerted immunosuppressive effects through the reduction of pro-inflammatory cytokine levels in our *in vitro* LPS model, iNOS expression remained unaffected. Previous studies have reported that iNOS induction in astrocytes under inflammatory conditions involves multiple signaling pathways beyond NF-κB (Ko et al., 2018; Saha and Pahan, 2006). We speculate that the observed result may be attributed to the ligand-bound AhR preferentially interacting with NF-κB-dependent signaling pathways that promote cytokine gene upregulation during the initial phase of LPS-induced neuroinflammation, while leaving other regulatory pathways that modulate iNOS-enhanced neuroimmunology responses in astrocyte cells unaffected.

Acute systemic administration of LPS has been documented in multiple studies to elicit behavioral alterations in mice, with motor activity and weight loss being the most significantly impacted (Biesmans et al., 2013; Sorrenti et al., 2018). In our study, similar behavioral changes were observed following LPS injection and deletion of AhR globally worsen the observed behavioral changes. Motor-related brain regions demonstrate enhanced glial cell inflammatory response following a single LPS injection (Carregosa et al., 2024). Thus, the diminished exploratory locomotor activity observed in AhRKO mice may be attributed to their heightened glial inflammatory response impacting the motor cortex. This study focused solely on the morphological alterations of microglia and astrocytes, as well as cytokine production in the hippocampus; however, future research examining the response of glial cells in AhRKO mice to LPS in the motor cortex is also necessary. Anxiety-like behaviors were also displayed by AhRKO mice, as these mice spent reduced time spent in the center of the open field box; however, it is premature to draw definitive conclusions, as further investigations employing additional anxiety-related behavioral tests are need to corroborate these observations. With regards to memory, a single dose of LPS in this study had no effect. Numerous studies have indicated memory deficits in mice administered LPS, utilizing the Y-maze, Novel Object test, or Morris water maze memory test (Ganesan et al., 2024; Morimoto et al., 2023; Schirmbeck et al., 2023); however, those studies either employed a chronic LPS injection model or assessed memory deficits at a later time point than in the current study.

Neuroinflammation is pivotal in the initiation of various neurodegenerative disorders, and integrates a complex interplay between resident immune cells in the brain, and the peripheral immune system (Maurya et al., 2024; Zang et al., 2022). Because loss of AhR seems to predispose both peripheral and central immune cells to heightened immunological responses, targeting AhR may be an intriguing therapeutic strategy to mitigate neuroinflammatory processes in various brain diseases. Also, considering that astrocytes and microglia interact in a bi-directional, cooperative manner to respond to pathological stimuli, augmenting AhR signaling in astrocytic cells may facilitate the maintenance or restoration of brain homeostasis by promoting neuronal survival and synaptic integrity, especially during the initial phases of ongoing inflammation. Future research focused on elucidating the role of AhR signaling in glial cells during chronic inflammatory states will be crucial for understanding the therapeutic potential of targeting AhR in neuroinflammatory disorders.

## Data Availability

The original contributions presented in the study are included in the article/supplementary material, further inquiries can be directed to the corresponding author/s.
